# An Intelligent Bearing Fault Transfer Diagnosis Method Based on Improved Domain Adaption

**DOI:** 10.3390/e27111178

**Published:** 2025-11-20

**Authors:** Jinli Che, Liqing Fang, Qiao Ma, Guibo Yu, Xiaoting Sun, Xiujie Zhu

**Affiliations:** Shijiazhuang Campus, Army Engineering University of PLA, Shijiazhuang 050003, China; 17603200861@163.com (J.C.); 15373828766@163.com (Q.M.); oec_ygb@163.com (G.Y.); 18134286885@163.com (X.S.); sduzxj@163.com (X.Z.)

**Keywords:** bearing fault diagnosis, domain adaptation, maximum mean discrepancy, transfer learning

## Abstract

Aiming to tackle the challenge of feature transfer in cross-domain fault diagnosis for rolling bearings, an enhanced domain adaptation-based intelligent fault diagnosis method is proposed. This method systematically combines multi-layer multi-core MMD with adversarial domain classification. Specifically, we will extend alignment to multiple network layers, while previous work typically applied MMD to fewer layers or used single core variants. Initially, a one-dimensional convolutional neural network (1D-CNN) is utilized to extract features from both the source and target domains, thereby enhancing the diagnostic model’s cross-domain adaptability through shared feature learning. Subsequently, to address the distribution differences in feature extraction, the multi-layer multi-kernel maximum mean discrepancy (ML-MK MMD) method is employed to quantify the distribution disparity between the source and target domain features, with the objective of extracting domain-invariant features. Moreover, to further mitigate domain shift, a novel loss function is developed by integrating ML-MK MMD with a domain classifier loss, which optimizes the alignment of feature distributions between the two domains. Ultimately, testing on target domain samples demonstrates that the proposed method effectively extracts domain-invariant features, significantly reduces the distribution gap between the source and target domains, and thereby enhances cross-domain diagnostic performance.

## 1. Introduction

Rotating machinery is widely used in large manufacturing systems and important technical equipment, such as ships and marine, wind turbines, aerospace engines, etc. [1,2]. Representing important transmission components of rotating machinery, rolling bearings play a key role in the stable operation of the entire rotating machinery system [3,4]. Therefore, fault diagnosis and the health assessment of rolling bearings are important [5,6].

Fault feature extraction plays a critical role in fault diagnosis. Since they mainly rely on subjective experience of experts, traditional fault extraction methods suffer poor generalization and global feature extraction capability. With the development of sensors and big data technology, large amounts of rolling bearing operation data are easy to obtain which inspires data-driven intelligent diagnosis to be a new trend. Data-driven intelligent diagnosis methods are mainly combined with deep learning networks such as CNN, LSTM, GRU, etc., to reveal fault information concealed in the big data. Since we use deep-learning networks rather than manual methods to extract time-frequency features from fault signals, those data-driven intelligent diagnosis methods experience high accuracy of fault diagnosis [7,8,9,10]. The progress in recent years has further enhanced the fault diagnosis based on deep learning through transformer structure [11,12], attention mechanism [13,14], and data expansion diffusion model [15,16]. The performance of processing complex fault modes under noise conditions is proved. Additionally, all the above fault diagnosis models are implemented based on the following assumptions: (1) labeled fault data are available, and (2) the training set and test set sample data have the same probability distribution. For practical fault diagnosis, the following problems make it difficult to satisfy these two conditions [17,18]: (1) Labeled fault data are difficult to obtain from some machines because the machines are not allowed to run to fault. (2) Labels are not available for practical fault diagnosis. (3) Due to variations in the environment, operating conditions, bearing quality, etc., these reasons reduce the generalization ability of cross-domain diagnostic models.

As an alternative to supervised learning approaches, anomaly detection-based methods have been developed to address the challenge of limited labeled fault data. The anomaly detection paradigm typically operates by learning the normal operating patterns of machinery using only healthy state data and subsequently identifying deviations from this learned baseline as potential anomalies or faults. This approach has the significant advantage of not requiring labeled data from damaged machines, which can be difficult or costly to obtain in practice. Various anomaly detection techniques, including statistical methods, one-class support vector machines, and autoencoder-based deep learning methods, have been successfully applied to bearing fault diagnosis. However, these methods face challenges in distinguishing between different fault types and may produce false alarms under varying operating conditions. Therefore, while anomaly detection provides a valuable complementary approach, methods capable of cross-domain knowledge transfer remain essential for scenarios requiring precise fault classification under changing conditions.

The above problems can be effectively solved by transfer learning, which focuses on solving some unknown but related diagnostic tasks in the target domain through the diagnostic knowledge learned from the source domain. The data in the target domain contain relevant diagnostic knowledge, but its distribution is different from the source domain. Currently, transfer learning methods in fault diagnosis mainly contain four classes of [17] GAN-based methods, instance-based methods, parameter fine-tuning-based methods, and feature transfer-based methods. Among them, the GAN-based method trains the diagnostic model by using the generator to generate fake labeled data similar to the target domain, and its transfer performance depends on the quality of the data generated by the generator [19]. The latest development in the past two years has introduced the diffusion model [16,20] as an alternative to the traditional Gan, providing more stable training and higher quality synthetic fault data generation to solve the problem of data imbalance. The instance-based method is easy to achieve in practical applications, but the method cannot be applied to transfer situations in which the data have serious discrepancy, and this diagnostic model lacks a strong data fitting capability [21]. The parameter fine-tuning-based approach focuses on pre-training the model using samples from the source domain, saving the pre-trained model parameters, and assigning them to the diagnostic model in the target domain. Finally, the parameters of the diagnostic model are fine-tuned by a small number of labeled samples in the target domain. Finally, the diagnostic performance of these two methods depends on the number of labeled samples in the target domain, which has some limitations in practical application [22].

Because of the problems existing in the above methods, the feature-transfer-based method completes the cross-domain diagnosis task by establishing an intelligent diagnosis model and uses the feature extraction network to extract the deep distribution features of the data. In this method, the distribution discrepancy of features is calculated by distance metric, and the parameters of feature mapping are updated by minimizing the objective function, which is helpful to reduce the distribution discrepancy of samples and extract the maximum domain-invariant features in the network. This method is less affected by the number of samples in the target domain and is suitable for scenarios with serious distribution differences. Therefore, the feature-based transfer methods have been widely studied [23,24,25].

In the domain adaptive diagnosis methods, according to the way of calculating the discrepancy between the sample distribution of the source domain and the target domain, they can be divided into the adversarial-based methods and the distribution discrepancy metrics methods.

(1) At present, in the field of cross-domain fault diagnosis, the adversarial-based diagnosis method classifies the target domain samples by adding a domain classifier after the feature extraction network. Ganin et al. proposed a domain adversarial neural network (DANN) and trained the network through a gradient reversal layer [26]. Wang et al. introduced DANN into the bearing fault diagnosis to achieve the purpose of completing the diagnosis under the transfer task of different loads [27]. It was also verified that DANN can provide competitive results in the limited available training time [27]. Li et al. constructed a new deep convolutional residual feature network to extract features and used the source domain labeled samples and the target domain unlabeled samples for domain adversarial training to improve the transmission performance of the diagnostic model [28]. The above methods can effectively reduce the training time by using domain classifiers to train on samples in the source domain and target domain, but this method lacks the representation of the feature information of the input domain and will have the defect of single network feature extraction.
(2) In cross-domain fault diagnosis, the diagnostic method based on distribution discrepancy metrics uses MMD to reduce the difference in feature distribution between samples in the source domain and the target domain. For example, by employing a single kernel MMD as a domain distance loss, Tzeng et al. proposed the deep domain confusion model (DDC) to obtain the discrepancy of the features which are, respectively, learned from the source domain and target-domain at the highest layer of the feature extraction networks [29]. To reduce distribution discrepancy and inter-class distance of transferable features learned by the network, Lei et al. construct FTNN to minimize multi-layer and multi-kernel MMD (ML-MK MMD) between source domain and target domain, leading to learning transferable features more efficiently [30]. Although it achieves some degree of transfer learning ability, the DDC suffers the drawback of merely learning single layer feature related to fault data. ML-MK MMD with better domain adaptive ability may be an option for handling this problem.
(3) To solve the problems of domain shift and single extracted features, related researchers combined MMD with domain classifier loss. Guo et al. proposed a Deep Convolutional Transfer Learning Network (DCTLN)-based approach that combines a single-kernel MMD with domain classifier loss to construct a new loss function that maximizes domain-invariant features across domains by training the network [31]. Yao et al. constructed a pair of pseudo-Siamese feature extractors by constructing two network-based feature extractors with the same structure but unshared parameters, and combined MMD and unbalanced adversarial training algorithms to train a domain adaptive network to accomplish cross-domain fault diagnosis [32]. However, the above methods only use the combination of single-layer MMD and domain classifier loss to construct a new loss function to train the network. This method only calculates the MMD of the highest layer of the network but ignores the discrepancy in the feature distribution of other layers in the network, which leads to the problem that the extracted high-level features in the network are insufficient. In conclusion, the first problem in the above methods is the insufficient extraction of features by the feature transfer network. The second problem is that there is a discrepancy in the cross-domain sample distribution. To address the aforementioned challenges, Fast Fourier Transform (FFT) is initially employed to preprocess the input signal and extract its frequency-domain features. Subsequently, the multi-layer multi-kernel maximum mean discrepancy (ML-MK MMD) is utilized to quantify the distributional differences in features between the source and target domain samples. This approach enables the feature extraction network to thoroughly extract maximal domain-invariant features. To further reduce the feature distribution discrepancy between the source and target domains, the ML-MK MMD is integrated with a domain classifier loss during training to construct a novel loss function. This new loss function is used to train the network, effectively minimizing the discrepancy in feature distributions between the source and target domains.

The remainder of the article is structured as follows. Section 2 presents the relevant theoretical background. Section 3 details the enhanced domain adaptive diagnosis method proposed. Section 4 provides the experimental validation of the proposed method. Section 5 concludes with a summary and future outlook.

## 2. Theoretical Background

### 2.1. Transfer Learning

In the context of transfer learning, we first introduce the general mathematical notation. Domain D consists of a feature space X and a marginal probability distribution P(X), where X = {*x*_1_, *x*_2_, …, *x*_n_}∈X. Task T comprises a label space Y and a conditional probability distribution P(Y|X), which can be learned from the training data. In this paper, subscript s denotes the source domain and subscript t denotes the target domain.

The transfer learning problem is then described as follows: The source domain with labeled data and its learning task are represented by $D_{s}$ and $T_{s}$, respectively, while the target domain without labeled data and its learning task are represented by $D_{t}$ and $T_{t}$, respectively. In general, $D_{s}\neq D_{t}$ and $T_{s}\neq T_{t}$, one should employ the learning task $T_{s}$ to exploit diagnostic knowledge concealed in the source domain $D_{s}$, leading to the ability to diagnose faults related to the target domain $D_{t}$ to enhance it. In addition, the transfer learning framework of this paper is based on the following assumptions.

(1) The source and target domains are related but exhibit distinct data distributions.
(2) The fault diagnosis tasks remain consistent across domains, with shared class labels.
(3) Labeled data from the source domain are utilized to train the network model.
(4) Unlabeled data from the target domain are employed for both training and testing of the network model.

Let X be an input space and $Y=\left\{1,2,\ldots ,I_{c}\right\}$ be a set of fault classes. $D_{s}={\left\{\left(x_{i}^{s},y_{i}^{s}\right)\right\}}_{i=1}^{n_{s}}$ represents the set of the source domain with $n_{s}$ labeled data in which $x_{i}^{s}$ and $y_{i}^{s}$ represent the data and its labels, respectively, while $D_{t}={\left\{\left(x_{i}^{t}\right)\right\}}_{i=1}^{n_{t}}$ represents the set of target domain with $n_{t}$ unlabeled data in which it represents the data. In addition, the marginal probability distributions of $D_{s}$ and $D_{t}$ are P(X), $Q\left(X\right)$ respectively, where P(X)≠Q(X).

A deep transfer learning model is proposed, which aims to classify data in the target domain by learning invariant features that are hidden across different domains.

### 2.2. Question Description

Traditional fault diagnosis is based on known fault types to identify the location, type, and extent of faults. It is commonly assumed that the sample distributions in the source and target domains are identical, enabling the fault diagnosis knowledge learned from the source domain to be directly applied to the unlabeled test samples in the target domain. However, the above assumptions are not applicable in practical transfer learning. Therefore, a cross-domain diagnosis method based on feature transfer is introduced.

A summary and analysis reveal that existing transfer learning methods are confronted with the following issues:

1. An unavoidable domain shift exists between the source and target domains, resulting in a distribution discrepancy that must be addressed, and most of the existing methods choose to add a domain classifier to reduce the domain distribution discrepancy; however, this method lacks an adequate feature representation of the input signals, which will lead to poor cross-domain diagnosis ability and poor generalization performance of the trained diagnostic model.
2. In terms of feature extraction, most of the existing methods use a single-layer, single-kernel MMD, and the use of this method to train the network will lead to the network having the defect of insufficiently extracting features and not being able to adequately extract cross-domain maximized domain-invariant features. To address the aforementioned challenges, an enhanced domain adaptation method for cross-domain fault diagnosis is introduced. To overcome the limitation of single feature extraction, ML-MK MMD is utilized to measure the distribution discrepancy between the source and target domains. Furthermore, a feature extraction network is designed to extract comprehensive domain-invariant features. To mitigate the domain shift issue, a novel loss function is formulated by integrating ML-MK MMD with the domain classifier loss. This strategy effectively optimizes the objective function, reducing the distribution discrepancy between the source and target domain samples, and ultimately enabling accurate cross-domain fault diagnosis.

### 2.3. Maximum Mean Discrepancy (MMD)

By supposing the probability distributions of the dataset $X={\left\{x_{i}\right\}}_{i=1}^{n_{1}}$ and $Y={\left\{y_{i}\right\}}_{i=1}^{n_{2}}$ are p and q, respectively. Let H denote a Reproducing Kernel Hilbert Space (RKHS) associated with a positive definite kernel function k: χ×χ→ℝ, where χ is the input feature space. The distribution discrepancy between the source and target domains is quantified by employing multi-kernel maximum mean discrepancy (MK-MMD) [33], as detailed in (1):

(1) $$D_{H}(X,Y):=\underset{\Phi \in H}{\sup}\left[E_{X\sim p}[\Phi (x)]-E_{Y\sim q}[\Phi (y)]\right]$$

where $D_{H}(X,Y)$ stands for maximum mean dispersion between distributions X and Y in RKHS. The larger the index, the greater the distribution difference. The supremum of the output set is denoted as sup(⋅), where H refers to the Reproducing Kernel Hilbert Space (RKHS) and $\Phi \left(\cdot \right)$ represents the mapping function that maps features from the original space into the RKHS. The RKHS is a Hilbert space of functions equipped with an inner product, where point-wise evaluation is a continuous linear function. The key advantage of using RKHS is that, through the kernel trick, we can compute inner products in high-dimensional space without explicitly constructing the mapping φ, i.e., ⟨φ(*x*), φ(*x*′)⟩ℋ = k(*x*, *x*′). This property enables efficient computation of distribution discrepancy even in infinite-dimensional feature spaces. In practice, the true distributions p and q are unknown, and we only have access to finite samples from each domain. Therefore, we use an empirical estimate of MMD. The empirical estimate of the MMD is then expressed by the following Equation (2):

(2) $$\begin{array}{l} {\overset{\wedge }{D}}_{H}^{2}\left(X,Y\right)={\left\|\frac{1}{n_{1}}\sum_{i=1}^{n_{1}}\Phi (x_{i})-\frac{1}{n_{2}}\sum_{j=1}^{n_{2}}\Phi (y_{i})\right\|}_{H}^{2}\\ =\frac{1}{n_{1}^{2}}\sum_{i=1}^{n_{1}}\sum_{j=1}^{n_{1}}k_{\sigma a}(x_{i},x_{j})-\frac{2}{n_{1}n_{2}}\sum_{i=1}^{n_{1}}\sum_{j=1}^{n_{2}}k_{\sigma a}(x_{i},y_{j})\\ +\frac{1}{n_{2}^{2}}\sum_{i=1}^{n_{2}}\sum_{j=1}^{n_{2}}k_{\sigma a}(y_{i},y_{j}) \end{array}$$

This empirical estimator has been proven to be unbiased and consistent [33], ensuring reliable quantification of distribution discrepancy between domains. Where $n_{1}$ and $n_{2}$ denote the number of training samples in the source and target domains, respectively. Where $x_{i}$ and $x_{j}$ represent individual samples of sets X and Y. $K_{\sigma \sigma }(x_{i},x_{j})$ represents the kernel function used to evaluate the similarity between $x_{i}$ and $x_{j}$. σ represents the bandwidth parameter of the Gaussian (RBF) kernel, and the subscript σσ represents the bandwidth used by the kernel. ${\left\|\cdot \right\|}_{H}$ represents the norm in the Reproducing Kernel Hilbert Space (RKHS) and $k_{\sigma i}\left(x_{i},x_{j}\right)=\sum_{a=1}^{N_{k}}k_{\sigma i}\left(x_{i},x_{j}\right)$ defines the feature kernel. The choice of Gaussian kernels is motivated by several theoretical and practical considerations:

(1) Universal approximation property: Gaussian kernels are universal kernels [26], meaning that the RKHS they induce is dense in the space of continuous functions. This ensures that MMD with Gaussian kernels can distinguish between any two different probability distributions, making it a powerful metric for detecting distribution discrepancies.
(2) Smoothness and differentiability: Gaussian kernels are infinitely differentiable, which is crucial for gradient-based optimization during neural network training.
(3) Interpretable bandwidth parameter: The bandwidth σ controls the scale at which distribution differences are measured. Small bandwidths capture local differences, while large bandwidths capture global distributional shifts. Since some MMDs with different kernels are employed to act on RHKS, Gaussian kernels with distinct bandwidth parameters are selected as the feature kernels for MMD.

## 3. Proposed Method

### 3.1. Framework

In this section, we present the proposed Improved Domain Adaptive Intelligent Diagnosis Method (IDAIDM). The objective is to develop an intelligent cross-domain diagnostic model based on a feature-based transfer learning approach. The framework of IDAIDM comprises the following four modules: the domain partition module, the feature extraction module, the domain adaptation module, and the fault identification module.

Comparison with joint distribution alignment is as follows: Joint adaptation network [34] uses joint MMD (JMMD) to align the joint distribution of features and labels. However, this requires source domain labels during alignment, which may be overfit to source specific patterns. IDAIDM aligns edge feature distributions and uses adversarial training to achieve label independent domain obfuscation. Comparison with conditional/class conditional methods is as follows: CDAN [35] conditionally applies domain classifiers to predicted class labels, which may be effective but introduces complexity in gradient computation. We found that for bearing fault diagnosis with limited training epochs, a simpler unconditional adversarial method combined with multi-layer MMD provides better stability and comparable or superior performance. The key advantage of IDAIDM is the following collaborative combination: multi-layer MMD explicitly reduces distribution differences at multiple scales, while adversarial training implicitly encourages domain obfuscation. Multi-layer alignment is more comprehensive than single-layer methods and has higher computational efficiency than methods that require joint distribution estimation.

**Figure 1:**
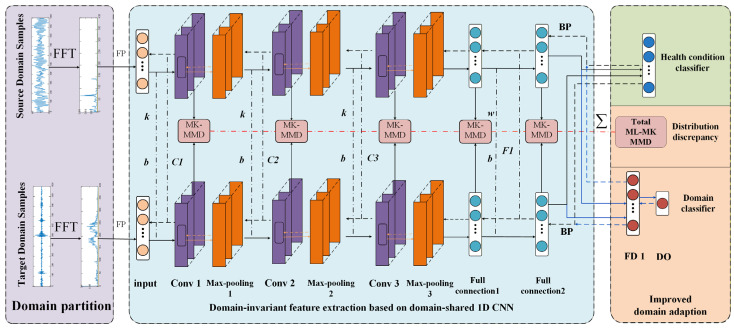
Architecture of the domain-shared 1D-CNN feature extraction network in the proposed IDAIDM.

The overall framework of the model is illustrated in Figure 1. Within the domain partition module, the dataset is divided into source and target domains, where the source domain contains labeled samples, while the target domain consists of unlabeled samples. Subsequently, samples from both domains undergo FFT, and the transformed frequency-domain signals are fed into the network.

In the feature extraction module, transferable features are extracted from labeled samples in the source domain and unlabeled samples in the target domain using a domain-shared one-dimensional CNN network that applies nonlinear feature mapping. The source and target domain data are processed using the same one-dimensional CNN architecture, with shared weights maintained across both domains.

The domain adaptation module consists of domain classifier loss and distribution discrepancy metrics, leveraging ML-MK MMD to compute the high-level feature distribution discrepancy across network layers. The domain classifier calculates domain classification loss for samples from both the source and target domains, where domain labels (source = 0, target = 1) are assigned based on the origin of the samples rather than their fault types. This enables domain classification to be performed on unlabeled target domain samples, aiding the 1D-CNN in learning domain-invariant features. The computed results from the domain adaptation module and the classifier loss are jointly utilized as the network’s overall optimization objective. The parameters of the nonlinear feature mapping are trained through backpropagation. The optimization objective is to minimize both the distribution discrepancy and the domain classification loss associated with the transferable features, thereby achieving maximized domain-invariant features with minimal cross-domain discrepancy.

The last part is the fault identification module, which feeds the unlabeled target domain samples into the network, and the category classifier can realize the correct classification of the unlabeled samples in this domain.

#### 3.1.1. Domain Partition Module

This module consists of samples from both the source and target domains, where the source domain samples are labeled, while the target domain samples remain unlabeled. In this module, preprocessing of samples from both domains is required. Since varying loads cause differences in the distribution of vibration data, Fast Fourier Transform (FFT) is employed to extract frequency-domain features from the bearing vibration signal as a preprocessing step [36]. While more advanced techniques such as envelope analysis (envelope FFT) are often recommended for bearing fault diagnosis to better extract modulated fault characteristic frequencies [37], the FFT provides a baseline frequency-domain representation that, when combined with deep learning, enables the 1D-CNN to automatically learn hierarchical and discriminative features through its multi-layer architecture. The preprocessed frequency-domain signal is then fed into the feature extraction network as input. The effectiveness of this approach is demonstrated by the high cross-domain diagnostic accuracy achieved in the experiments, where the combination of FFT preprocessing and deep domain adaptation successfully handles varying load conditions.

#### 3.1.2. Feature Extraction Module

The feature extraction module extracts features from different domain samples by building a domain-shared CNN. The module is achieved by a one-dimensional CNN which has ten layers, including one input layer, three convolutional layers, three pooling layers, two fully connected layers, and one output layer. The structural parameters of the one-dimensional CNN are shown in Table 1. The input signal is the frequency domain signal after FFT. The input layer is followed by the convolution layer in which the input data and the convolution kernel are operated by convolution to learn the features of the input signal. Since the input signal is one-dimensional vibration data in the time domain, a one-dimensional convolutional kernel is used to extract temporal features from the signal. The convolution operation slides along the time axis to detect local patterns and anomalies. The forward operation of each convolutional layer is expressed as:

(3) cj=ReLU(∑i=1nwc∗sj−m+1:ji+bc)

The detailed meaning is to take the convolution kernel $w_{c}\in R^{m}$ and convolve it with the j-th fragment signal $s_{j-m+1}^{i}\in R^{m}$ to extract the features. * is the symbol of one-dimensional convolution, $w_{c}$ is the convolution kernel, $b_{c}$ is the corresponding deviation,n is the number of convolution kernels, and $c_{j}$ is the output of the j-th convolution layer. The size of the convolution kernel of convolution layer 1 is 64, the step size is set to 1, and the padding is 64, where the size of the convolution kernel of convolution layers 2 and 3 is 5, the step size is 1, and the padding is 2. ReLU is chosen as the activation function after the convolution layer.

After each convolutional layer, it is connected to a pooling layer, where pooling operations are applied to reduce the dimensionality of the convolutional features. The max-pooling function is employed, and its expression is as follows:

(4) $$p_{j}=\max\left\{c_{j\times k:(j+1)\times k}\right\}$$

k is the length of the pooling and $P_{j}$ is the pooling output of the j-th sample. The kernel size of the pooling layer is 2 with a step size of 2.

Following each convolutional layer, a pooling layer is incorporated to perform downsampling, thereby reducing the dimensionality of the extracted convolutional features. The max-pooling function is utilized.

After the convolutional pooling operation, it is flattened by fully connected layers 1, 2, and 3:

(5) $$f=\sigma ({\left(w_{f}\right)}^{T}s_{m}+b_{f})$$

$w_{f}$ denotes the weight matrix of the fully connected layer, $b_{f}$ represents the corresponding bias vector, and $S_{m}$ refers to the input data. The final classification layer employs the Softmax function to estimate the category of the output data. The specific formulation is as follows:

(6) $$y=\frac{1}{\sum_{i=1}^{K}f_{i}}\left[\begin{array}{l} f_{1}\\ f_{2}\\ \cdot \\ \cdot \\ \cdot \\ f_{K} \end{array}\right]$$

$f_{K}$ denotes the output of the second fully connected layer (FC2), where K represents the number of distinct fault classes. y represents the probability distribution output of the Softmax layer.

#### 3.1.3. Domain Classification Module

The domain classification module is set after the fully connected layer 2, as illustrated in Figure 1; the domain classifier comprises two layers: a fully connected layer FD1 and a domain discriminative output layer DO, where FD1 is connected to the second fully connected layer in 1D-CNN. The parameters of the domain classifier structure are shown in Table 2. The DO output layer of the domain classifier is a binary classifier with logistic regression, which uses logistic regression to predict the probability of samples belonging to the source domain and its expression is as follows:

(7) $$d=\frac{1}{1+e^{-({\left(w_{d}\right)}^{T}f_{FD1}+b_{FD1})}}$$

where $d(x)\in \left[0,1\right]$ denotes the predicted probability that input sample *x* belongs to the source domain (d(*x*) = 1 indicates source domain, d(*x*) = 0 indicates target domain), gi denotes the *i*-th sample’s feature vector output from the FD1 layer, where $i\in \left\{1,2,\ldots ,n\_batch\right\}$ and n_batch are the batch size, $w_{d}$ denotes the weight matrix of the domain classifier module, $b_{FD1}$ denotes the corresponding bias vector, and $f_{FD1}$ denotes the mapping function of the first fully connected layer (FD1) in the domain classifier, which transforms the 512-dimensional input from FC2 to a 256-dimensional intermediate representation.

The domain classifier serves a dual purpose in the proposed method:

(1) Domain discrimination: The classifier attempts to distinguish whether an input sample originates from the source domain (label = 0) or the target domain (label = 1) based on the learned features.
(2) Adversarial feature learning: Through the Gradient Reversal Layer (GRL) mechanism, the domain classifier engages in adversarial training with the feature extractor. Specifically, the domain classifier tries to correctly classify domain labels (minimize its classification error). The feature extractor tries to fool the domain classifier (produce features that make domain classification difficult). When the domain classifier cannot accurately distinguish between source and target domain samples, this indicates that the feature extractor has successfully learned domain-invariant features. These domain-invariant features contain information relevant to fault types while being insensitive to domain shifts caused by different operating conditions.

#### 3.1.4. Improvement of the Domain Adaptive Module

The transferability of features learned from cross-domain data is influenced by the distribution discrepancy between domains. In existing studies, such as the Deep Domain Confusion (DDC) method [22], distribution discrepancy is typically computed only at the highest fully connected layer, based on the recognition that high-level features at this layer are critical for transfer learning. However, this single-layer approach (i.e., computing MMD only at the top layer) has a limitation: the domain-invariant features learned by the network tend to be less diverse [26], as they ignore distribution discrepancies that exist in intermediate layers. In contrast, multi-layer approaches that compute distribution discrepancy across multiple network layers have been shown to learn more transferable features efficiently [23].

To address this limitation, an improved domain adaptive method is proposed. In this method, instead of computing distribution discrepancy only at the last fully connected layer, the calculation is extended to multiple layers of the network. Specifically, in addition to the last fully connected layer, the calculation of distribution discrepancy is incorporated into the convolutional layers C1, C2, and C3, as well as an additional fully connected layer. This integration aims to adjust the distribution of transferable features at multiple levels within the network. The parameters of the domain-shared convolutional neural network (CNN) are optimized by combining the joint multi-layer multi-kernel maximum mean discrepancy (ML-MK MMD) loss with the domain classifier loss during the training process. convolutional layers (C1, C2, C3), and an additional fully connected layer. The ML-MK MMD of the learned transferable features is then calculated as follows:

(8) $$\begin{array}{c} D_{H}^{2}(Z^{L,s},Z^{L,t})=\frac{1}{n_{s}^{2}}\sum_{i=1}^{n_{s}}\sum_{j=1}^{n_{s}}\sum_{l\in L}k(x_{i}^{l,s},x_{j}^{l.s})\\ -\frac{2}{n_{s}n_{t}}\sum_{i=1}^{n_{s}}\sum_{j=1}^{n_{t}}\sum_{l\in L}k(x_{i}^{l,s},x_{j}^{l.s})\\ +\frac{1}{n_{t}^{2}}\sum_{i=1}^{n_{t}}\sum_{j=1}^{n_{t}}\sum_{l\in L}k(x_{i}^{l,t},x_{j}^{l.s}) \end{array}$$

where $D_{H}^{2}$ is the square of the maximum mean square calculated by kernel H. $Z^{L,s}$ and $Z^{L,t}$ are the multilayer transfer features in the source and target domains, respectively. $x_{i}^{l,s},x_{j}^{l.t}$ represent the eigenvectors of the *i*-th source domain sample and the *j*-th target domain sample in layer L, respectively. $n_{s}$ and $n_{t}$, respectively, represent the number of samples in the source domain and the target domain. $L=\left\{C_{1},C_{2},C_{3},F_{1},F_{2}\right\}$ is the index of each layer.

The domain-shared CNN facilitates the learning of transferable features with specific attributes, while the ML-MK MMD dynamically adjusts throughout the training process. In (6), the bandwidth and time complexity of the kernel can seriously affect the MMD. In this paper, the Gaussian kernel, whose expression is shown below, is selected:

(9) $$k(x_{i}^{s},x_{i}^{t})=\exp(-{\left\|x_{i}^{s}-x_{i}^{t}\right\|}^{2})/2y^{2}$$

where y represents the bandwidth of the Gaussian kernel. As shown in Equation (9), the discrepancy in the distribution of transferable features learned during training is ultimately achieved by minimizing the sum of the multilayer MMDs, which can be expressed by the following equation:

(10) $$L_{ml-mmd}=\underset{\theta^{L}}{\min}{\overset{\wedge }{D^{2}}}_{H}(Z^{L,s},Z^{L,t})$$

Among them, $Z^{L,s},Z^{L,t}$ represent the feature sets L of the source and target domains on the layer, respectively. $\theta^{L}$ is the set of parameters for layers $C_{1}$, $C_{2}$, $C_{3}$, $C_{4}$.

### 3.2. Optimization Objective

IDAIDM contains three optimization objectives, which are combined to construct a total loss function for network training. The three optimization objectives are as follows:

(1) Minimize the fault classification loss Lc for labeled samples in the source domain (Equation (11)). This loss is only computed on source domain samples because only they have fault labels.
(2) Maximize the domain classification loss Ld for samples from both source and target domains (Equations (12) and (13)). This loss uses domain labels (source/target) rather than fault labels and can be computed for both domains.
(3) Minimize multilayer MMD for transferable features learned from both source and target domain datasets (Equation (10)). This is a distribution-based metric that does not require labels.

#### 3.2.1. Classification Loss

Since the labeled training samples are available, the first optimization objective is to minimize the classification error of the training samples. In this paper, the cross-entropy function is used as the loss function for the set of sample sets in the source domain, which is defined as follows:

(11) $$L_{cla}=-\frac{1}{n}\sum_{i=1}^{n}{\left(y_{i}^{s}\right)}^{T}\cdot \log(h_{i}^{F_{2},s})$$

where n is the size of the batch size of the network training samples, $h_{i}^{F_{2},s}$ is the probability distribution of the label of the output samples of the source domain in layer $F_{2}$, and $y_{i}^{s}$ is the corresponding label of the samples of the source domain.

#### 3.2.2. Domain Classification Loss

During the training of the domain-shared network, the sample from the source domain with labels and the sample from the target domain without labels are fed into the network, and the samples from both domains are classified by a domain classifier in terms of domain categories. If the domain classifier cannot discriminate features between the source and target domains, the features are proved to be domain-invariant. Therefore, the third optimization objective of this network is to maximize the domain classification loss in the data from the source domain to the target domain. The domain classification loss is defined as:

(12) $$L_{a}=\frac{1}{m}\sum_{i=1}^{m}\left(g_{i}\log d\left(x_{i}\right)+(1-g_{i})\log(1-d(x_{i}))\right)$$

where $g_{i}$ is the true domain label, $d(x_{i})$ is the domain output of the i-th sample,$x_{i}$ is the sample from the source and target domains.

In the training phase, the training dataset consists of $n_{s}$ source domain samples and $n_{t}$ target domain samples. Thus, Equation (12) can also be written as the following:

(13) $$L_{adv}=\frac{1}{n_{s}}\sum_{i=1}^{n_{s}}L_{a}(f{\mathrm{FD}}_{i}^{\left(S\right)})+\frac{1}{n_{t}}\sum_{j=1}^{n_{t}}L_{a}(f{\mathrm{FD}}_{i}^{\left(T\right)})$$

where $f{\mathrm{FD}}_{i}^{\left(S\right)}$ and $f{\mathrm{FD}}_{i}^{\left(T\right)}$ represent the high-level features learned from the source domain data and the target domain data in the FD layer, respectively.

#### 3.2.3. ML-MK MMD Loss and Total Loss Function

The third optimization objective is to minimize the distribution discrepancy between the source and target domains across multiple network layers.

In order to efficiently transfer the trained classifier from the source domain to the target domain, the two domain distributions of the input data are brought closer in the representation of the learned features. By minimizing $L_{ml-mmd}$, the source and target domains are closer in the learning representation, so that a network for extracting domain invariant features of fault-related information is insensitive to domain transfer at the same time. Having defined the three individual optimization objectives—classification loss (Equation (11)), domain classification loss (Equation (13)), and ML-MK MMD loss (Equation (10))—the total loss function of IDAIDM is formulated by combining these three components:

(14) $$L_{total}=L_{cla}-\lambda L_{adv}+\beta L_{ml-mmd}$$

where λ, β are weight parameters that determine the weight shares of the domain classifier and multilayer MMD, respectively. GRL (Gradient Reversal Layer) is introduced in the network to realize the transformation of the gradient so that the network can be backpropagated during training. Therefore, in $L_{total}$, there is a negative sign in front of $L_{adv}$. In $L_{total}$ the trade-off parameters λ and β are varied from 0 to 1 by means of 2/(1+exp(−10×p))−1; p is a linear parameter that accompanies the number of Epoch from 0 to 1 [24] where *p* represents the training progress, defined as *p* = e/E, in which e is the current epoch number and E is the total number of training epochs. As training progresses from epoch 1 to E, the parameter *p* increases linearly from 0 to 1, gradually increasing the influence of both the domain classification loss and the ML-MK MMD loss. The network is trained by minimizing $L_{total}$ as the optimization objective.

### 3.3. Training Process

IDAIDM is trained by the SGD optimizer for $L_{total}$. The loss function of the network is minimized by training, and the total loss function including the parameters of each module can be expressed by the following equation:

(15) $$L_{total}=\underset{\theta_{f},\theta_{c},\theta_{d}}{\min}L_{cla}(\theta_{f},\theta_{c})-\lambda L_{adv}(\theta_{f},\theta_{d})+\beta L_{ml-mmd}(\theta_{f})$$

$\theta_{f}$, $\theta_{c}$, and $\theta_{d}$ are the sets of parameters for the feature extraction network, the fault type classifier, and the domain classifier, respectively. The method updates the parameter sets in the network by the SGD, and the expressions for each part of the parameter update are shown below:

(16) $$\theta_{f}\leftarrow \theta_{f}-\mu (\frac{\partial L_{cla}}{\partial \theta_{f}}-\lambda \frac{\partial L_{adv}}{\partial \theta_{f}}+\beta \frac{\partial L_{ml-mmd}}{\partial \theta_{f}})$$

(17) $$\theta_{c}\leftarrow \theta_{c}-\mu (\frac{\partial L_{cla}}{\partial \theta_{c}})$$

(18) $$\theta_{d}\leftarrow \theta_{d}-\mu (\frac{\partial L_{adv}}{\partial \theta_{d}})$$

Among them, $\partial_{L}/\partial_{\theta }$ represents the partial derivative of the loss L relative to the parameter θ, which is calculated using the backpropagation algorithm. In Equation (16), the feature extractor receives gradients from all three targets, which are represented by $\partial L_{cla}/\partial \theta_{f}$ for reducing classification errors, $\partial L_{adv}/\partial \theta_{f}$ for increasing domain confusion (through GRL inversion), and $\partial L_{ml-mmd}/\partial \theta_{f}$ for aligning feature distributions. In Equation (17), the fault classifier only focuses on optimizing the source domain classification to minimize the cross-entropy loss of labeled samples. In Equation (18), the domain classifier learns to distinguish between the source and target through the GRL mechanism to counteract the feature extractor, where μ is the learning rate in the network.

**Figure 2:**
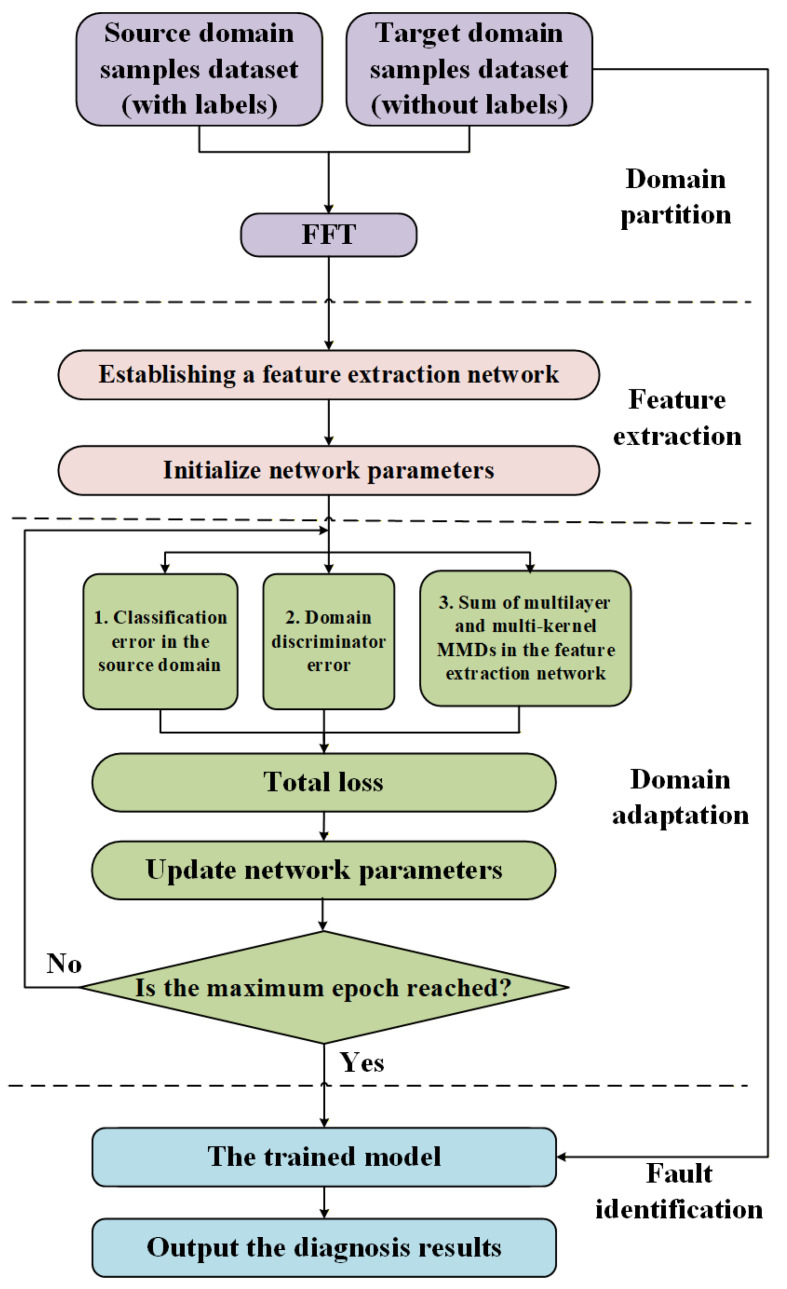
Flowchart of the training process of the IDAIDM.

The flow chart of the training process of the diagnostic model proposed in this paper is shown in Figure 2. In domain partition module, the collected datasets are processed, where the data sample set with labels is in the source domain and the data sample set without labels is in the target domain.

In the feature extraction step, each parameter of the network is first initialized, and then samples in the source and target domains are both input to the domain-shared CNN for forward propagation, thus learning layer by layer to obtain the transferable features between different domains.

In the domain adaptation module, the ML-MK MMD and the loss of the learned transferable features of domain classifier are calculated by Equations (10) and (13), respectively. Then, these two losses (Equations (10) and (13)) are combined with the classification loss in the source domain to obtain the total loss of the network. The SGD is used to back-propagate the network and update the parameters. The number of training epochs is set to train the diagnostic model proposed in this paper until the set judgment conditions are satisfied. After the conditions are satisfied, the diagnostic model is used in the fault identification step to input the set of target domain test samples without labels to achieve the diagnostic classification of target domain samples and complete the purpose of cross-domain fault diagnosis.

## 4. Experiment

### 4.1. Dataset Introduction

**Figure 3:**
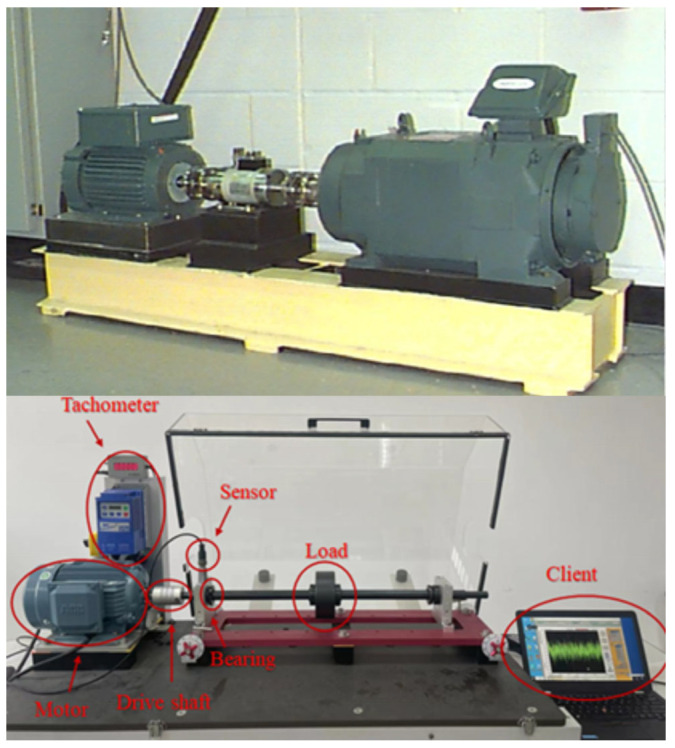
The test rig of the CWRU and Self-Built dataset.

To verify the diagnostic ability of the model in different scenarios, this paper conducted multiple experiments on two datasets. Two datasets are, respectively, from Case Western Reserve University (A, B, C) [38] and our research team (D, E, F, G). Their experimental equipment and specific operating conditions are shown in Figure 3 and Table 3, respectively. Among them, from top to bottom there are CWRU experimental equipment and our experimental equipment, respectively.

The CWRU bearing vibration data are collected by accelerometers at the drive end of motor. The single-point bearing fault was simulated in the laboratory by electric discharge machining. The CWRU bearing dataset consists of vibration signals with four health states, i.e., normal condition (NC), outer ring fault (OF), inner ring fault (IF), and ball fault (BF). In this experiment, the sample frequency was set to 12 kHz. In this experiment, the data from CWRU is used as shown in Table 3, and dataset A (motor speed is 1797 r/min) is collected without load, dataset B (motor speed is 1750 r/min) is collected under 2HP motor load, and dataset C (motor speed is 1730 r/min) is collected under 3HP motor load.

The Self-Built dataset includes the following five operating conditions: rotational speeds of 900 rpm, 1200 rpm, 1500 rpm, and 1800 rpm. The two types of loads are no-load applied and a load of 5 kg applied. The sampling frequency is 12 kHz.

In the entire text, the traditional and well-known CWRU dataset is mainly used as the main dataset to run all necessary experiments, while the Self-Built dataset is used as the auxiliary dataset to demonstrate the generalization and robustness of this method. In this experiment, samples from each dataset are pre-processed before input into the network, and the frequency domain features of the fault signal are extracted by FFT. There are 1000 training samples for each health state in datasets A, B, and C in the model training step, i.e., each dataset contains a total of 1000 × 4 training samples. Each health state in the test set contains 100 test samples, i.e., there are 100 × 4 samples to be tested in the test set. Detailed information is shown in Table 3. Each individual sample in each dataset contains 2048 sampling points, and pre-processed data are used for data enhancement with a sample length of 100 overlap. Each signal is subjected to FFT and since the transformed spectrum is symmetric, only half of the spectrum is retained, i.e., the number of feature points for each sample is 1024. The labels are set to 0, 1, 2, and 3 for different health states in the source domain dataset, respectively.

From Table 3, it can be seen that the experiment contains twelve transfer learning tasks (six for each of the two datasets), and the dataset A is denoted as the source domain with labels and the dataset B is denoted as the target domain without labels. For the CWRU dataset, this cross-domain task is denoted as A→B; furthermore, the other five transfer learning tasks in this experiment are B→A, B→C, C→B, A→C, and C→A. For the Self-Built dataset, all selected transfer learning tasks are D→E, D→F, D→G, F→G, E→D, and F→D. The purpose of establishing the tasks is to verify the cross-domain diagnosis performance of this method under different transfer tasks.

### 4.2. Parameters Selection

**Figure 4:**
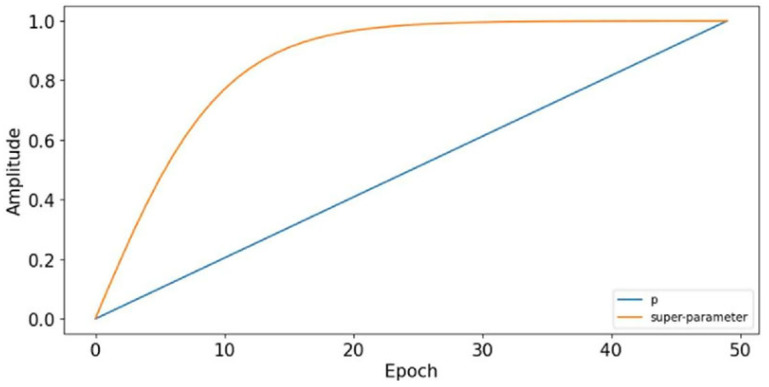
The trade-off parameter of the proposed method.

In this experiment, Epoch is set to be 50 and the change process of the trade-off parameter in the total loss function during the training process is shown in Figure 4. In the domain adaptation module, ML-MK MMD computes the high-level feature discrepancy between source and target domain training samples at different layers of the network by using a multi-kernel bandwidth RBF. Studies have shown that the simple bandwidth parameter and a mixture of five kernels have good experimental results in experiment [34]. Therefore, in this paper, the bandwidth parameters are chosen as 1, 2, 4, 8, and 16 and their weights are kept equal. During training, the batch size is set to 128 and the SGD optimizer with a learning rate of 0.0001 is used for training to make the total loss function converge to a minimum. The structure and parameters of the network are kept constant in the following experiments.

In this experiment, the total number of training epochs is set to E = 50. Figure 4 shows the evolution of the following two key parameters during training: (1) the training progress parameter *p* (blue line), defined as *p* = e/E where e is the current epoch, which increases linearly from 0 to 1; and (2) the adaptive trade-off parameter λ(*p*) (orange line), which follows the schedule λ(p)=2/(1+exp(−10×p))−1 and controls the weight of the domain classification loss in Equation (14). As training progresses, λ(*p*) increases from 0 to approximately 1 following a sigmoid-like curve, gradually increasing the influence of adversarial domain adaptation.

### 4.3. Results

**Figure 5:**
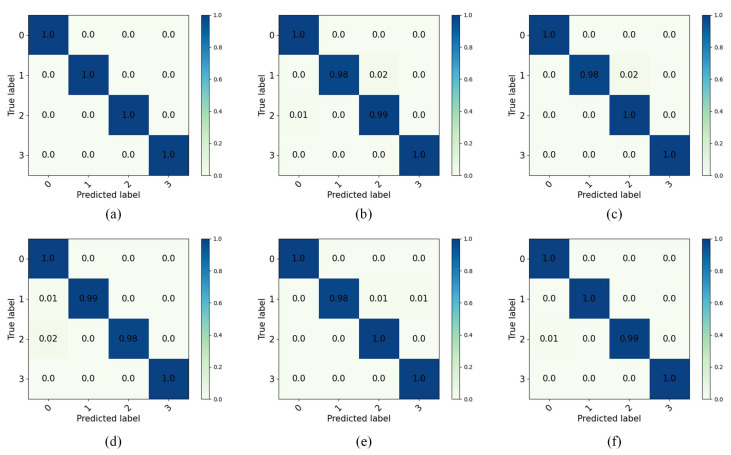
Confusion matrices of target domain test results for all six cross-domain transfer tasks in the CWRU dataset. (**a**) Task A→B, (**b**) Task A→C, (**c**) Task B→C, (**d**) Task B→A, (**e**) Task C→A, (**f**) Task C→B.

**Figure 6:**
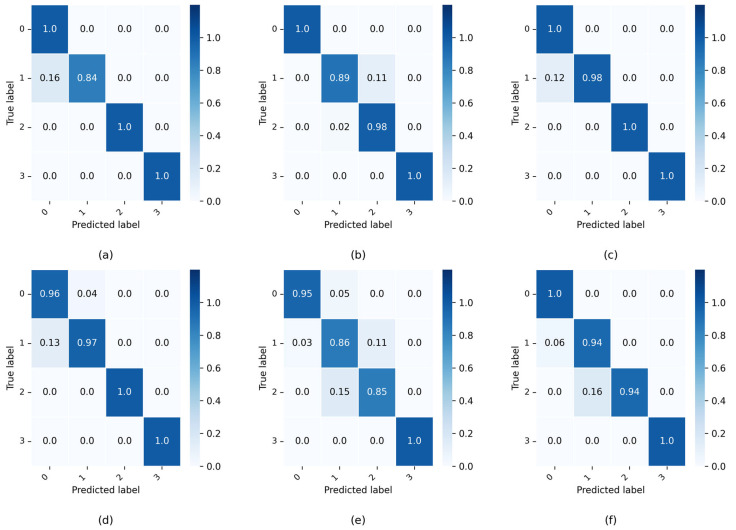
Confusion matrices of target domain test results for all six cross-domain transfer tasks in the Self-Built dataset. (**a**) Task D→E, (**b**) Task D→F, (**c**) Task D→G, (**d**) Task F→G, (**e**) Task E→D, (**f**) Task F→D.

After setting each parameter in the transfer diagnostic model, the network training epoch is set to 50, 1000 × 4 source domain samples with labels, 1000 × 4 target domain samples without labels from each task are input to the diagnostic model for training, and the network is trained with the SGD optimizer. To avoid the chance of training results, the model is trained 10 times under each task. After training, 100 × 4 target domain test samples without labels are input to the network for predictive classification, and the classification result accuracy is shown by confusion matrix. In the CWRU dataset of this experiment, the accuracy of the predicted classification results for the target domain test samples under six different tasks is shown in the confusion matrix, and the final test results are presented in Figure 5. In the Self-Built dataset, the final test results are shown in Figure 6.

Figure 5 shows that the IDAIDM has good diagnostic results for NC and OF and its prediction accuracy reaches 100% in six tasks, i.e., it is able to accurately classify samples of this fault type when 1000 samples are input. By comparing the six different transfer tasks, it is found that IF and BF are misclassified in their diagnostic results. The diagnostic result of Task A→B is the best with 100% test accuracy for all of them, and the test accuracy of the rest of the tasks is above 98%. Figure 6 shows that IDAIDM exhibits excellent diagnostic performance for NC and OF in the Self-Built dataset, achieving 100% prediction accuracy in multiple transmission tasks when processing input samples. Through comparative analysis of six different transfer tasks, it was observed that BF and IF exhibited a certain degree of misclassification in the diagnostic results. The diagnostic results of task D→E showed the most consistent performance and almost perfect accuracy in most fault categories, while the testing accuracy of all tasks remained above 92%. The analysis shows that under different transfer tasks, the recognition accuracy of IDAIDM on the target domain test samples is also different, and the effect in extracting domain-invariant features is also different.

The confusion matrix results show that the model is more effective in transferring for NC and IF compared to BF and OF. The experimental results show that the method can effectively classify the test samples, which verifies the effectiveness of the proposed method in this paper. To visualize the quality of the learned feature representations, t-SNE (t-Distributed Stochastic Neighbor Embedding) is employed to project the high-dimensional features to a 2D space. Specifically, for each transfer task, the 512-dimensional feature vectors from the second fully connected layer (FC2, as shown in Table 1) are extracted for all 400 target domain test samples (100 samples per fault type). These features are then reduced to 2D coordinates using t-SNE while preserving the local structure of the feature space. Figure 5 shows the t-SNE visualization results for different transfer tasks, where each point represents a test sample and different colors indicate different fault types (0: NC, 1: IF, 2: BF, 3: OF).

**Figure 7:**
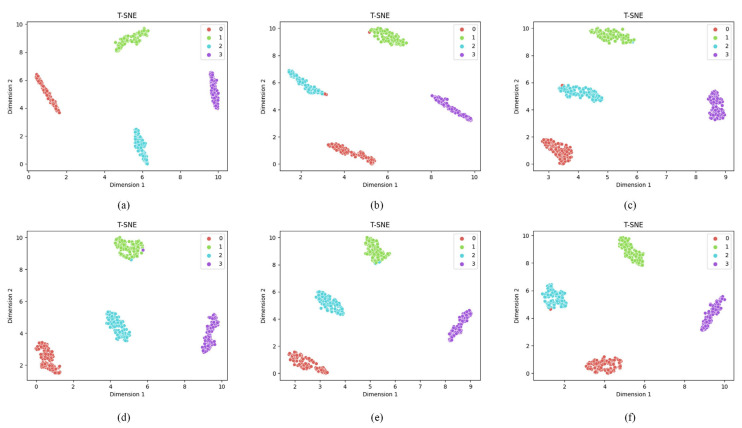
T-SNE of the transfer results of the target domain test samples under different transfer tasks. (**a**) Task A→B, (**b**) Task A→C, (**c**) Task B→C, (**d**) Task B→A, (**e**) Task C→A, (**f**) Task C→B.

Figure 7 presents the t-SNE visualization results for all CWRU six transfer tasks, revealing the quality of learned feature representations and the effectiveness of domain adaptation. Each sub-figure is analyzed as follows:

(a) Task A→B: The feature distributions show excellent clustering with clear separation between all four fault classes (NC, IF, BF, OF). The clusters are compact and well-separated, indicating perfect domain adaptation, which is consistent with the 100% accuracy shown in Figure 5a. This task demonstrates the best transfer performance among all scenarios.
(b) Task A→C: The visualization reveals good overall clustering, with NC and BF forming distinct, well-separated clusters. However, slight overlap is observed between IF (class 1, red points) and OF (class 3, purple points) in the feature space, explaining the minor misclassifications observed in the confusion matrix. Despite this, the majority of samples remain correctly clustered.
(c) Task B→C: Similar to Task A→C, this scenario shows clear separation for NC and BF, while a small number of IF and OF samples exhibit proximity in the feature space, leading to occasional misclassifications. The overall clustering structure remains intact, indicating effective but not perfect domain adaptation.
(d) Task B→A: The t-SNE plot demonstrates good clustering quality for NC and OF, with both classes forming tight, well-separated clusters. However, some IF and BF samples show partial overlap, resulting in minor misclassifications. The majority of samples from each class remain correctly grouped.
(e) Task C→A: This task shows moderate clustering performance. While NC maintains clear separation from other classes, there is noticeable overlap between IF, BF, and OF in certain regions of the feature space. This explains the slightly lower accuracy compared to Task A→B, though the model still achieves acceptable classification performance.
(f) Task C→B: The visualization indicates good separation for NC and reasonable clustering for other fault types. Some IF and OF samples appear close in the feature space, consistent with the minor misclassifications observed in the confusion matrix.

Overall, Figure 7 demonstrates that the proposed IDAIDM successfully learns domain-invariant features that cluster samples of the same fault type together while maintaining inter-class separation. Across all tasks, the following patterns are observed: (1) NC (normal condition) consistently forms the most distinct cluster with minimal confusion with fault classes; (2) OF (outer race fault) generally shows good separability; (3) IF (inner race fault) and BF (ball fault) occasionally exhibit overlap in challenging transfer scenarios, suggesting these fault types have more similar vibrational characteristics under certain operating conditions. The decreasing intra-class distance and increasing inter-class distance validate the effectiveness of the multi-layer multi-kernel MMD and adversarial domain adaptation strategies employed in IDAIDM.

### 4.4. Comparisons with Other Methods

**Figure 8:**
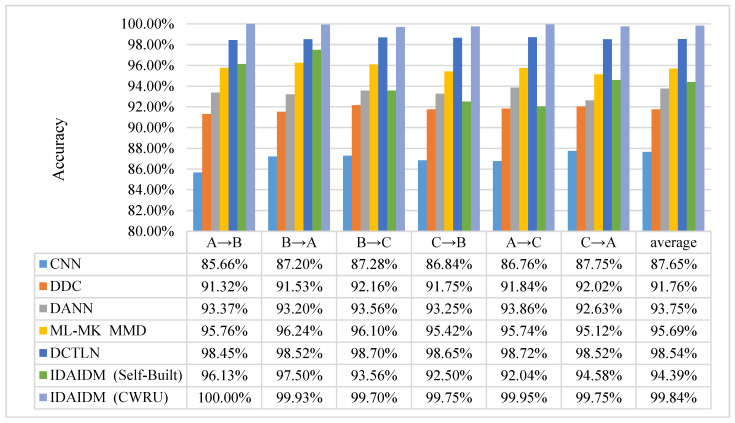
Bearing fault diagnosis results of different methods under different transfer tasks.

To validate the effectiveness of IDAIDM, it is compared with five other migration methods across six different migration tasks. These methods include Convolutional Neural Network (CNN), Deep Domain Confusion Network (DDC), Domain Adversarial Training Model (DANN), Multi-Layer Domain Adaptive Network Model (ML-MK MMD), and Deep Convolutional Translational Learning Network (DCTLN). Among them, CNN is a standard one-dimensional convolutional neural network with the same architecture as our feature extractor but only trained on source domain data without any domain adaptation mechanism. This serves as a baseline to demonstrate the necessity of transfer learning. DDC uses single core MMD loss at the highest fully connected layer (FC2) to minimize domain differences. DANN uses gradient reversal layer (GRL) and domain classifier to learn domain invariant features through adversarial training. This architecture matches our network, but without MMD loss. DCTLN combines domain classifiers with single-layer single kernel MMD. This architecture uses the same feature extractor and domain classifier as ours but only uses a single kernel to compute MMD at FC2. The differences between the compared methods are shown in Table 4. In order to ensure the accuracy of the experiment, the parameters in each model of the above method in different transfer tasks are kept the same as the selection in this method, and the training samples are input into the above models. After training is completed, the target domain test samples are input into each model for testing and the average of the 10 test results is taken as the final result as shown in Figure 8.

Figure 8 shows the diagnosis results of IDAIDM and other methods. The results show that IDAIDM outperforms the other five transfer learning methods in six different cross-domain fault diagnosis tasks. From the table it can be seen that the average diagnostic accuracy of IDAIDM in six cross-domain fault diagnosis tasks in the CWRU dataset is 99.84% and the accuracy of this model in Task A→B for the target domain test samples reaches 100.00%, while the accuracy of the other five methods is above 99.00%, which is better than the other five methods. Table 4 shows that this paper uses ML-MK MMD in the domain adaptive module in combination with loss of the domain classifier to minimize the total optimization objective of the construction, fully extract the domain-invariant features, and reduce the feature distribution discrepancy. The experiment results show that IDAIDM in this paper is better than other methods, and the accuracy is higher than other methods. This shows the effectiveness and superiority of IDAIDM in different cross-domain tasks.

**Figure 9:**
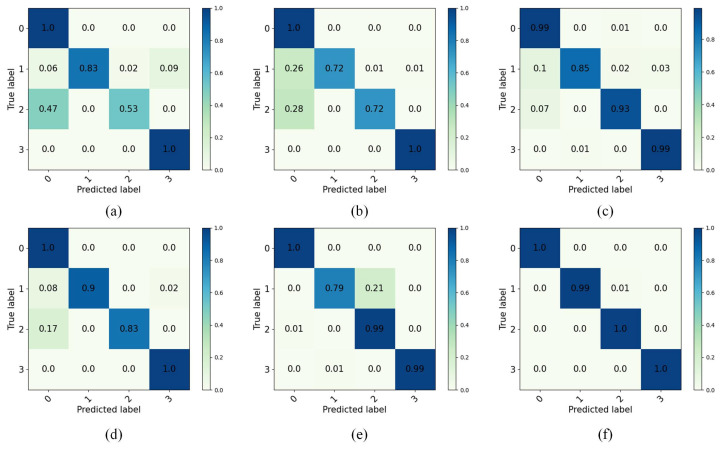
Confusion matrix of sample transfer results for different methods in the target domain under Task C→B. (**a**) CNN, (**b**) DDC, (**c**) DANN, (**d**) ML-MK MMD, (**e**) DCTLN, (**f**) IDAIDM.

In order to show the classification accuracy between each method in the test set for different fault types, the classification of predicted and true labels for each fault type after testing are shown by the confusion matrix. Without loss of generality, among the six different fault diagnosis learning tasks, the classification results of Task C→B are selected for illustration. The results of the six aforementioned diagnosis methods for the classification of the 400 test set samples in Task C→B are shown in Figure 9.

In Figure 9, the classification accuracy of IDAIDM is observed to be higher than that of the other five methods for the test set in Task C→B. IDAIDM has a good effect on the classification of NC, IF, BF, and OF, and its recall rate can reach 100%, and the recall rate of IF is 99%. Among the four types of faults in Table 5, the transfer features of NC and OF are easy to extract and their accuracy is higher in classification, while the accuracy of IF and BF is lower. This result indicates that fault features of IF and BF are more difficult to transfer in cross-domain fault diagnosis tasks.

**Figure 10:**
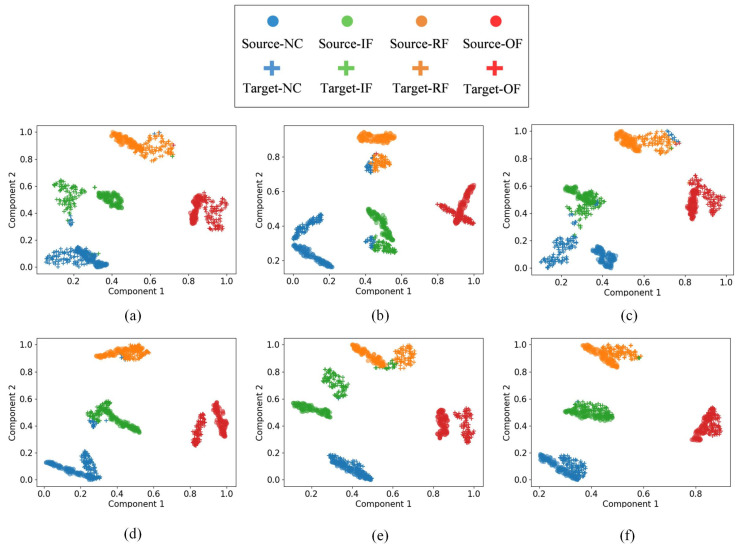
TSNE of different methods in Task C →B: (**a**) CNN, (**b**) DDC, (**c**) DANN, (**d**) ML-MK MMD, (**e**) DCTLN, (**f**) IDAIDM.

For Task C→B, we extract features from both source domain (dataset C) and target domain (dataset B) test samples after training. Figure 10 shows the t-SNE visualizations for the six compared methods. In each subplot, points from the source domain and target domain are plotted together, with different colors representing different fault types and different markers distinguishing source and target domains. In Figure 10, Figure 10a–f show the results of the above six methods, and it can be seen from the above figures that IDAIDM has a better clustering effect for all four faults compared with the other methods, and the results show that IDAIDM has a better domain confusion capability.

However, for the mixed transfer features, the proposed method has the best clustering results compared with other methods, which helps to solve the classification problem of the target domain samples. In Figure 10a–f, it can be found that IDAIDM has smaller intra-class distance and larger inter-class distance with significantly less cross-domain discrepancy. The proposed method not only effectively adapts the distribution of learned transferable features but also expands the inter-class distance of learned transferable features. As a result, they are easily distinguished in cross-domain diagnosis with small distribution discrepancy, and this result intuitively gives the reason for the better diagnostic performance of the present model over other methods.

To further explain the importance of this module and comprehensively evaluate the effectiveness of the proposed IDAIDM method, we conducted ablation experiments. The experimental design aims to address the key research question of what individual contributions each component in the framework makes. All experiments were trained and tested on CWRU under the same conditions to ensure fair comparison. As shown in Table 6 below, the training process employs the SGD optimizer with a learning rate of 1 × 10^-4^, and each experiment is repeated five times with different random seeds to ensure statistical significance.

The ablation study provides crucial insights into the contribution of individual components. The results unequivocally demonstrate that both the domain discriminator and ML-MK MMD are indispensable for optimal performance. Notably, the removal of ML-MK MMD leads to a more substantial performance degradation (8% average drop) compared to removing the domain discriminator (4% average drop), indicating the fundamental importance of statistical moment matching in domain alignment.

## 5. Conclusions

This paper proposed an improved domain adaptive intelligent diagnosis method (IDAIDM) to address the challenge of cross-domain bearing fault diagnosis under varying operating conditions. The method integrates multi-layer multi-kernel maximum mean discrepancy (ML-MK MMD) with adversarial domain adaptation to extract comprehensive domain-invariant features across multiple network layers.

The experimental results on the CWRU dataset demonstrate the effectiveness of the proposed approach. The main results and conclusions are as follows:

(1) IDAIDM achieved an average diagnostic accuracy of 99.84% across six different cross-domain transfer tasks (A→B, A→C, B→A, B→C, C→A, C→B), significantly outperforming five baseline methods including CNN (87.65%), DDC (91.76%), DANN (93.75%), ML-MK MMD (95.69%), and DCTLN (98.54%).
(2) The t-SNE visualizations confirm that IDAIDM successfully learns features with smaller intra-class distances and larger inter-class distances, indicating effective extraction of domain-invariant and discriminative features.
(3) The combination of ML-MK MMD with adversarial training through the domain classifier provides complementary benefits: MMD explicitly minimizes distribution discrepancy through feature statistics, while the domain classifier implicitly learns domain-invariant representations through adversarial training.

The proposed method makes several contributions to the field of transfer learning-based fault diagnosis. It addresses the limitation of existing methods that rely solely on single-layer distribution alignment or adversarial training alone. The multi-layer, multi-kernel approach enables more comprehensive feature alignment, resulting in improved generalization to new operating conditions without requiring labeled data from the target domain.

Future work should explore several directions:

(1) validating the method on more complex industrial datasets with greater variability in operating conditions and noise levels.
(2) investigating the integration of more advanced signal processing techniques, such as envelope analysis, with the domain adaptation framework.
(3) extending the method to handle scenarios with different fault types between source and target domains (partial domain adaptation).

In conclusion, the proposed IDAIDM method demonstrates strong performance in cross-domain bearing fault diagnosis, offering a promising approach for intelligent maintenance systems that must operate reliably across varying conditions.

For the convenience of readers, we first provided a complete semantic table of various acronyms at the end of the article, as shown in Table 7 below.

## Figures and Tables

**Table 1:** The architecture of the one-dimensional CNN.

Layers	Parameters	Stride	Padding	Activation Function	Input Size	Output Size
Input	/	/	/	/	/	(1 × 1024)
Conv1	(1,16,64)	1	32	ReLU	(1 × 1024)	(16 × 1024)
Max pooling1	2 × 1	2	0	/	(16 × 1024)	(16 × 512)
Conv2	(16,32,5)	1	2	ReLU	(16 × 512)	(32 × 512)
Max pooling2	2 × 1	2	0	/	(32 × 512)	(32 × 256)
Conv3	(32,64,5)	1	2	ReLU	(32 × 256)	(64 × 256)
Max pooling3	2 × 1	2	0	/	(64 × 256)	(64 × 128)
Flatten	0	/	/	ReLU	(64 × 128)	8192
FC1	(128 × 64,1024)	/	/	/	8192	1024
FC2	(1024,512)	/	/	/	1024	512
FC3	(512,Class)	/	/	Softmax	512	Class

**Table 2:** The architecture of the domain classifier.

Layers Categories	Parameters	Output Size
FD1	(512,256)	256
D0	(256,1)	1

**Table 3:** Introduction to CWRU and Self-Built datasets.

Datasets	Fault Categories	Labels	Number of Samples	Operation Conditions
A	NC	0	1000	(0HP) 1797 r/min
	IF	1		
	BF	2		
	OF	3		
B	NC	0	1000	(2HP) 1750 r/min
	IF	1		
	BF	2		
	OF	3		
C	NC	0	1000	(3HP) 1730 r/min
	IF	1		
	BF	2		
	OF	3		
D	NC	0	1000	(0HP) 1800 r/min
	IF	1		
	BF	2		
	OF	3		
E	NC	0	1000	(1HP) 1800r/min
	IF	1		
	BF	2		
	OF	3		
F	NC	0	1000	(0HP) 2400 r/min
	IF	1		
	BF	2		
	OF	3		
G	NC	0	1000	(1HP) 2400 r/min
	IF	1		
	BF	2		
	OF	3		

**Table 4:** Differences of different methods.

Methods	FFT	Domain Classifier	Single-Kernel MMD	ML-MK MMD	Accuracy
CNN	√				87.65%
DDC	√		√		91.76%
DANN	√	√			93.75%
ML-MKMMD	√			√	95.69%
DCTLN	√	√	√		98.54%
IDAIDM (Self-Built)	√	√		√	94.39%
IDAIDM (CWRU)	√	√		√	99.84%

**Table 5:** Recall rate of task C→B for 4 types of different methods.

Methods	NC	IF	BF	OF
CNN	1.00	0.83	0.53	1.00
DDC	1.00	0.72	0.28	1.00
DANN	0.99	0.85	0.93	0.99
ML-MK MMD	1.00	0.90	0.83	1.00
DCTLN	1.00	0.79	0.99	0.99
IDAIDM	1.00	0.99	1.00	1.00

**Table 6:** Performance comparison of different model schemes.

Transfer Task	Complete Method	w/o Discriminator	w/o ML-MK MMD
A→B	1.00	0.94	0.92
A→C	0.99	0.95	0.88
B→C	0.99	0.94	0.92
B→A	0.99	0.96	0.91
C→A	0.99	0.96	0.93
C→B	0.99	0.96	0.92
Average	0.99	0.95	0.91
Performance Drop	/	−0.04	−0.08

**Table 7:** Alphabet abbreviation semantic table.

Acronyms and Abbreviations	Complete Semantics
BF	Ball Fault
CDAN	Conditional Domain Adversarial Network
CNN	Convolutional Neural Network
CWRU	Case Western Reserve University
DANN	Domain Adversarial Neural Network
DCTLN	Deep Convolutional Transfer Learning Network
EMD	Empirical Mode Decomposition
FC	Fully Connected Layer
FD	Fully Connected Layer in Domain Classifier
FFT	Fast Fourier Transform
FTNN	Feature Transfer Neural Network
GAN	Generative Adversarial Network
GRL	Gradient Reversal Layer
HP	Horsepower
IDAIDM	Improved Domain Adaptive Intelligent Diagnosis Method
IF	Inner Ring Fault
JAN	Joint Adaptation Network
JMMD	Joint Maximum Mean Discrepancy
LSTM	Long Short-Term Memory
ML-MK MMD	Multi-Layer Multi-Kernel Maximum Mean Discrepancy
NC	Normal Condition
OF	Outer Ring Fault
RKHS	Reproducing Kernel Hilbert Space
RMS	Root Mean Square
RNN	Recurrent Neural Network
SGD	Stochastic Gradient Descent
STFT	Short-Time Fourier Transform
t-SNE	t-Distributed Stochastic Neighbor Embedding

## Data Availability

The original contributions presented in this study are included in the article. Further inquiries can be directed to the corresponding author.
